# Preparation and Solution Properties of Zwitterionic Polyacrylamide for Enhancing Oil Recovery

**DOI:** 10.3390/molecules31122128

**Published:** 2026-06-17

**Authors:** Xiaobing Wei, Feng Li, Boyi Zhong, Jie Li, Yanling Xiao, Cuiqin Li

**Affiliations:** 1 College of Economic and Management, Northeast Petroleum University, Daqing 163318, China; dqweixiaobing@126.com; 2 Provincial Key Laboratory of Polyolefin New Materials, College of Chemistry and Chemical Engineering, Northeast Petroleum University, Daqing 163318, China; lifeng_dqpi@sina.com (F.L.); lijie838@163.com (J.L.); 3 College of Wildlife and Protected Area, Northeast Forestry University, Harbin 150040, China; zhongjiwe@126.com

**Keywords:** zwitterionic copolymer, polyacrylamide, segmentation initiation, enhance oil recovery

## Abstract

The viscosity stability of polymer solution is one of the challenges in enhancing oil recovery, and zwitterionic copolymers present excellent viscosity stability and emulsification performance, enabling effective control of the oil/water interface mobility and enhancing oil recovery. Herein, a zwitterionic copolymer (P(AM/AMBS/MAPTAC)) containing sulfonic acid groups and quaternary amine groups was synthesized by segmentation initiation with AM, AMBS and MAPTAC as monomers. The chemical structure of P(AM/AMBS/MAPTAC) was confirmed by FTIR and ^1^H NMR. The *M_w_* value of P(AM/AMBS/MAPTAC) was 9.91 × 10^6^ g/mol, and the apparent viscosity of the 2000 mg/L solution was 24.92 mPa·s at 60 °C at a salinity of 5000 mg/L. P(AM/AMBS/MAPTAC) with sulfonic acid groups and quaternary amine groups exhibited outstanding salt tolerance and shear resistance. When the salinity was 10,000 mg/L and the shear rate was 300 s^−1^, the apparent viscosity for the P(AM/AMBS/MAPTAC) solution was 23.45 mPa·s and the viscosity reduction rate was 69.23% at 60 °C for 30 d. Moreover, P(AM/AMBS/MAPTAC) exhibited an improved emulsifying property and a greater oil–water interface thickness than HPAM and SPAM due to the synergistic effect of the sulfonic acid and quaternary amine groups in the P(AM/AMBS/MAPTAC) molecule. The polymer flooding and alkali–surfactant–polymer flooding formed by P(AM/AMBS/MAPTAC) had high chemical oil recovery, and the oil displacement efficiency of P(AM/AMBS/MAPTAC) was higher than that of HPAM and SPAM in the polymer flooding and alkali–surfactant–polymer flooding systems.

## 1. Introduction

Polymer flooding has been used as an enhanced oil recovery (EOR) technique for a long time in many countries, and partially hydrolyzed polyacrylamide (HPAM) is a widely used polymer in polymer flooding and alkali–surfactant–polymer flooding due to its cost–effectiveness, good viscosity and physicochemical properties [1,2]. However, there are many drawbacks in the practical application of HPAM in a high–temperature and high–salinity reservoir [3]. Increasing the reservoir temperature leads to the breakdown of the molecular chain of HPAM, as well as a significant reduction in the solution viscosity. The thickening capability of HPAM in high-salt conditions is also significantly decreased [4], especially in the presence of Ca^2+^ and Mg^2+^. Many reports identified that the introduction of functional groups into the polyacrylamide backbone was favorable to improving the temperature tolerance and salt tolerance of polymers, and these polymers were used in harsh reservoir conditions, replacing HPAM [5,6,7,8,9,10]. Zhang [5] synthesized a terpolymer by introducing cationic monomers during the polymerization process of acrylamide and acrylic acid, and the synthesized cationic hydrophobically modified polyacrylamide had good viscosity stability in a solution of 2000 mg/L above 60 °C. Yang [9] prepared a novel amphiphilic polymer with hydrophobic monomer (C16DMAAC), acrylamide and acrylic acid as comonomers, and the amphiphilic polymer presented excellent viscoelasticity with enhanced aging stability and shear resistance. Moreover, the injection of the active amphiphilic polymer effectively improved the recovery of heavy oil. Wu [10] synthesized a salt–induced tackifying polymer (PAMNS) for enhancing oil recovery in high–salinity reservoirs, and PAMNS demonstrated superior performance in polymer flooding experiments under salinity ranges from 50,000 mg/L to 200,000 mg/L. Li [11] synthesized a kind of water–soluble sulfonate copolymer (SPAM) using acrylamide (AM), 2–(dimethylamino) ethyl methacrylate (DMAEMA) and 2–methyl–2–[(1–oxo–2–propenyl)amino]–1–propane sulfonic acid (AMPS) as monomers, and SPAM had good temperature tolerance and salt tolerance. Wang [12] synthesized a zwitterionic hydrophobically associating polymer using diallyl dimethyl ammonium chloride (DMDAAC), lauryl methacrylate (LAM), N,N–dimethylacrylamide (DMAM) and 2–acrylamido–2–methyl–1–propanesulfonic acid (AMPS) as monomers; the zwitterionic hydrophobically associating polymer had strong clay–particle adsorption and suspension stability as a rheology modifier and fluid loss reducer in water–based drilling fluids. However, reports on the emulsification and oil displacement performance of zwitterionic polyacrylamides under high–temperature and high–salinity conditions are relatively few so far. In addition, partially hydrolyzed polyacrylamides exhibit poor viscosity stability in high–temperature and high–salinity conditions, making them inadequate for harsh reservoir conditions.

Based on our previous work [11], this work attempted to study the viscosity stability and the oil displacement performance of a zwitterionic polyacrylamide in polymer flooding and alkali–surfactant–polymer flooding under high-temperature and high-salinity conditions. A zwitterionic polyacrylamide, P(AM/AMBS/MAPTAC), was synthesized by segmented initiation with 2,2′–azobis [2–methylpropionamidine] dihydrochloride and a redox initiation system. The effects of the copolymeric monomers and the polymeric parameters on the weight-average molecular weight (*M_w_*) and the solution viscosity were investigated in this work. The effects of temperature and salinity on the viscosity of the P(AM/AMBS/MAPTAC) solution were probed to investigate the viscosity stability under high-temperature and high–salinity conditions. The emulsification and oil displacement performance of P(AM/AMBS/MAPTAC) in polymer flooding and alkali–surfactant–polymer flooding were evaluated and compared with those of the commonly used partially hydrolyzed polyacrylamide (HPAM) and the sulfonate copolymer (SPAM) synthesized in our previous work [11]. This zwitterionic polyacrylamide could be a promising alternative to the widely used HPAM in chemical flooding. This work provides a new approach for developing zwitterionic polymers for oil recovery in high-temperature and high–salinity reservoirs.

## 2. Experiments

### 2.1. Materials and Instruments

Acrylic acid (AA), acrylamide (AM), 2–methyl–2–[(1–oxo–2–propenyl)amino]–1–propane sulfonic acid (AMPS), 2–acrylamide ethyl–4–methylbutanesulfonic acid (AMBS), 2–(dimethylamino) ethyl methacrylate (DMAEMA), 2′–azobis [2–methylpropionamidine] dihydrochloride (AIBA), ammonium persulphate (APS), N,N,N′,N′–tetramethylethylenediamine (TEMED), sodium formaldehyde sulfoxylate (HMSNa), sodium formaldehyde sulfoxylate, acrylic acid (AA), dimethylaminopropyl methacrylamide (DMAPMA), (3–acrylamidopropyl)trimethylammonium chloride (DAC), and (3–acrylamidopropyl) trimethylammonium chloride (MAPTAC) were purchased from Sigma–Aldrich (Shanghai, China). Sodium chloride (NaCl), sodium hydroxide (NaOH), magnesium chloride (MgCl_2_) and anhydrous calcium chloride (CaCl_2_) were purchased from Tianjin Kermel Chemical Reagent Co., Ltd. (Tianjin, China). Partially hydrolyzed polyacrylamide (HPAM, hydrolysis degree = 26%, *M_w_* = 2.5 × 10^7^ g/mol) was provided by Daqing Refining & Chemical Company (Daqing, China).

The FTIR spectrum of the copolymer was obtained using an FTIR 750 spectrometer (Nicolet Instrument Corp., Madison, WI, USA). The ^1^H NMR spectrum of the copolymer was measured on a 400 MHz spectrometer (Bruker400, Munich, Germany) with D_2_O as the solvent. The rheological properties were studied by a DHR–2 rotational rheometer (TA Instrument Corp., New Castle, DE, USA). The weight-average molecular weight (*M_w_*) of the copolymer was determined from the intrinsic viscosity [*η*] measurements by a Ubbelohde viscometer (Shanghai Yueping Scientific Instruments Co., Ltd., Shanghai, China) [11]. The hydrodynamic radius of the three polymer solutions at 2000 mg/L was measured at a salinity of 5000 mg/L and 60 °C with a ZS90 Dynamic Light Scattering instrument (Malvern Panalytical, Malvern, UK).

### 2.2. Preparation of Zwitterionic Polyacrylamide

A series of zwitterionic polyacrylamide copolymers were prepared in this work according to the synthesis method in our previous work [11]. Certain masses of AM and anionic monomer were dissolved in deionized water at 10 °C under a nitrogen atmosphere, and 10.0 wt% NaOH aqueous solution was added to the above solution to adjust the pH value of the solution to 8.0. AIBA solution was added to the above system, and the reaction mixture was stirred for 1 h at 25 °C under a nitrogen atmosphere. Then, an aqueous solution of cationic monomer and aqueous solutions of APS and HMSNa were added into the reaction mixture, and the mixture was stirred for 2 h at 10 °C under a nitrogen atmosphere. The reaction mixture was heated to the specified temperature and allowed to react for 8 h. The mixture was cooled to room temperature, and a solid was formed by adding a certain amount of ethanol into the mixture. The crude solid was treated with a mixture of N,N–dimethylformamide and acetic acid (1:1 by volume) to remove the unmodified polyacrylamide. The synthesized zwitterionic polyacrylamide was dried at 60 °C for 10 h in a vacuum oven.

### 2.3. Measurement of Rheological Properties

The rheological properties of the polymer solutions were studied with respect to the shear rate and salinity at 60 °C. The viscosity stability test was carried out at 60 °C for 30 d, and the viscosity reduction rate (*DR*) was calculated with Equation (1):

(1) $$\mathrm{DR}=\frac{\eta_{0}-\eta_{t}}{\eta_{0}}\times 100\%$$

where *η_t_* is the apparent viscosity of the polymer solution at 60 °C for 30 d (mPa·s), and *η*_0_ is the apparent viscosity of the polymer solution at 60 °C for 0 d.

### 2.4. Measurement of Emulsification

Emulsification was investigated via the bottle test method at 60 °C for 240 min. The simulated emulsion was prepared by mixing crude oil and the polymer solution (*V*_water_:*V*_oil_ = 4:1) in a homogenizer at a rotation speed of 10,000 r/min for 10 min. The emulsification efficiency (*DE*) of the polymers was calculated to evaluate the stability of the simulated emulsions according to Equation (2):

(2) $$\mathrm{DE}=\frac{V_{1}}{V_{0}}\times 100\%$$

where *V*_1_ is the volume of the separated water (mL), and *V*_0_ is the volume of the initial water (mL).

### 2.5. Core-Flooding Test

According to our previous work [11], cores (4.5 cm × 4.5 cm × 30 cm) were pretreated with 5% HCl solution and deionized water and dried under vacuum conditions. Then, simulated brine (NaCl, CaCl_2_ and MgCl_2_ mass ratio = 60:4:3) was continuously injected into the dried cores (4.5 cm × 4.5 cm × 30 cm) until a stable pressure was established to determine their permeability. The cores were saturated with simulated crude oil until no further water production occurred and were aged at 80 °C for 3 days. Simulated brine was continuously injected until the water cut reached 98%. After water flooding, polymer solution was injected to displace the simulated crude oil. The total oil recovery and the polymer recovery were calculated. The alkali–surfactant–polymer flooding test was similar to the polymer flooding test but with alkali–surfactant–polymer solution as the chemical oil-displacing agent.

### 2.6. Molecular Dynamics Simulation

According to our previous work [13], molecular dynamics simulations of P(AM/AMBS/MAPTAC) at the oil/water interface were conducted using Gromacs software (2020) [14] and Sobtop software (Version 1.0 [Dev 3.1]) [15], and n–undecane was used to model the oil phase. The simulation box consisted of a water film positioned between two oil layers, and two P(AM/AMBS/MAPTAC) molecules were dispersed at the oil–water interface using Packmol [16]. The P(AM/AMBS/MAPTAC) molecule used in the molecular dynamics simulations contained 35 acrylamide monomers + 5 AMBS monomers + 1 MAPTAC monomer, and the SPAM molecule contained 36 acrylamide monomers + 3 AMPS monomers + 1 DMAEMA monomer. All systems were enclosed within a box of dimensions 10 nm × 10 nm × 14 nm. The simulation was conducted at a temperature of 60 °C and a pressure of 0.1 MPa. The simulated equilibration period was 800 ps, and the total simulation period was 20 ns with a time step size of 2 fs.

## 3. Results and Discussion

### 3.1. Effect of Synthesis Conditions on the Properties of Zwitterionic Polyacrylamide

#### 3.1.1. Effect of Comonomers

**Figure 1:**
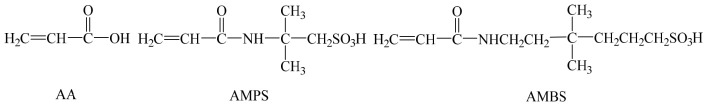
Chemical structures of anionic monomers.

When the molar concentration of AM was 0.3 mol/L, the molar ratio of AM to anionic monomer was 50:1, the molar ratio of AM to DMAEMA was 350:1, the AIBA concentration was 0.03 wt%, the concentration of the second initiator was 0.03 wt%, the reaction temperature was 50 °C, and the reaction time was 10 h, thse effects of the anionic monomer type (Figure 1) on the *M_w_* value of the zwitterionic polyacrylamide and the apparent viscosity of the 2000 mg/L copolymer solution at 60 °C were investigated at a salinity of 5000 mg/L (NaCl, CaCl_2_ and MgCl_2_ mass ratio = 60:4:3). The results are listed in Table 1.

As shown in Table 1, the *M_w_* value and the apparent viscosity of the zwitterionic polyacrylamide with AA as the anionic monomer were lower than those with the other anionic monomers. This is because, in contrast to AA, AMPS and AMBS contain alkyl groups and large sulfonic acid groups in their molecular structures. The steric hindrance effect of the alkyl groups and sulfonic acid groups in the side chain reduces the repulsion between the monomers, which is conducive to a copolymerization reaction to obtain a copolymer with a high *M_w_*. In addition, the polar groups in the molecules inhibit the curling of polymer chains during polymerization. According to Table 1, the apparent viscosity of the P(AM/AMBS/MAPTAC) solution with AMBS as the anionic monomer was higher than that of the solution using AMPS. However, the *M_w_* value of P(AM/AMBS/MAPTAC) was lower than that of the synthesized copolymer with AMPS as the anionic monomer. The higher apparent viscosity of P(AM/AMBS/MAPTAC) can be attributed to the enhanced intermolecular interactions induced by the specific polar groups and long alkyl chain in AMBS, which may promote molecular associations, facilitating the formation of a large molecular network in the solution. These results indicate that the type of anionic comonomer significantly influences both the molecular weight and the solution viscosity of a zwitterionic polyacrylamide. Although AMBS has a lower *M_w_* than AMPS, it shows potential advantages for improving viscosity.

**Figure 2:**
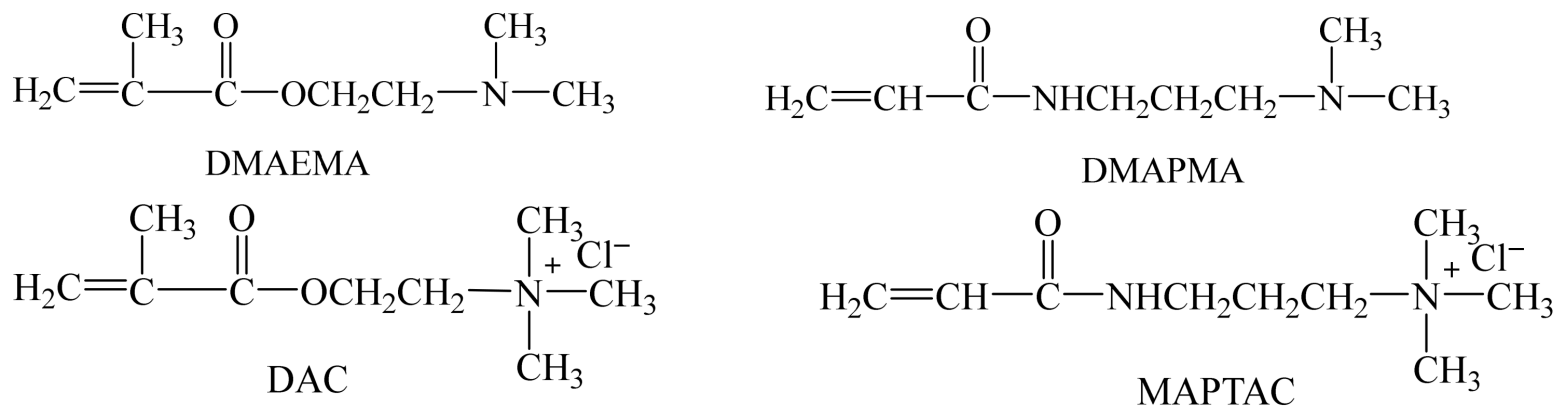
Chemical structures of cationic monomers.

Under the same test conditions as specified for Table 1, the effects of the cationic monomer type (Figure 2) on the *M_w_* value of the zwitterionic polyacrylamide and the apparent viscosity of the 2000 mg/L copolymer solution were investigated with AMBS as the anionic monomer; the results are listed in Table 2.

As shown in Table 2, the *M_w_* values of the synthesized copolymers with positively charged compounds as the cationic monomers were lower than those of the corresponding copolymers prepared with an uncharged compound as the cationic monomer. This phenomenon can be attributed to the differences in the charge density and steric hindrance of cationic monomers. DAC and MAPTAC, with higher charge density, tend to form stronger intermolecular electrostatic interactions, leading to the formation of more compact molecular aggregates during polymerization. These aggregates restrict chain growth to form a copolymer with low *M_w_*. In contrast, DMAEMA and DMAPMA have relatively lower charge density and greater steric hindrance from their side chains, which weakens the intermolecular associations and leads to the formation of a copolymer with high *M_w_*. However, the variation in the apparent viscosity followed an opposite trend to that of the *M_w_* values. The apparent viscosity is not only related to *M_w_* but also strongly influenced by the intermolecular interactions. The strong association between the positively charged segments of DAC and MAPTAC copolymers leads to the formation of molecular networks from aggregates in solution [17]. This network structure increases the flow resistance, thereby significantly increasing the apparent viscosity. Even though the *M_w_* of these copolymers is lower, the enhanced intermolecular associations dominate the viscosity behavior, resulting in higher apparent viscosity as compared to copolymers containing DMAEMA and DMAPMA. MAPTAC was used as the cationic monomer to synthesize the zwitterionic polyacrylamide (P(AM/AMBS/MAPTAC)) in this work.

#### 3.1.2. Effect of Polymeric Parameters

**Figure 3:**
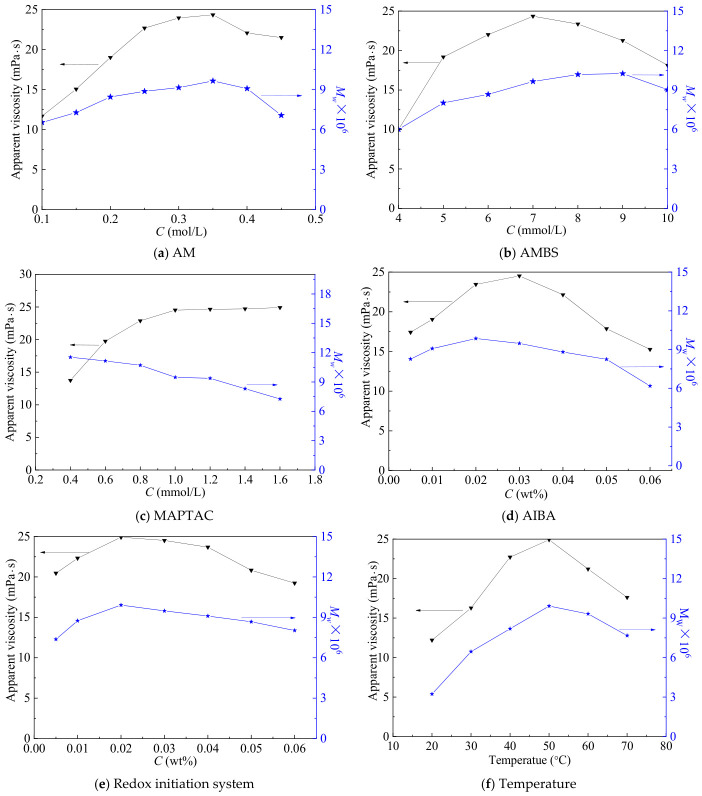
The effects of polymeric parameters on the *M_w_* of P(AM/AMBS/MAPTAC) and the apparent viscosity of the solution.

Based on the above results for the comonomers, the effects of the polymeric parameters on the *M_w_* value of P(AM/AMBS/MAPTAC) and the apparent viscosity of a 2000 mg/L polymer solution at 60 °C and a salinity of 5000 mg/L (NaCl, CaCl_2_ and MgCl_2_ mass ratio = 60:4:3) were investigated. The results are shown in Figure 3.

The AM and AMBS concentrations play an important role in the synthesis of P(AM/AMBS/MAPTAC). Both the *M_w_* value and the apparent viscosity of P(AM/AMBS/MAPTAC) first increased and then decreased with the AM and AMBS concentrations (Figure 3a,b). The *M_w_* value and apparent viscosity reached their maxima when the AM and AMBS concentrations were 0.35 mol/L and 7 mmol/L, respectively. At low monomer concentrations, increasing the monomer content in the polymerization system improved polymerization activity, which was favorable for forming a copolymer with high *M_w_*. The highest apparent viscosity of 23.96 mPa·s (Figure 3a,b) was obtained when the AM and AMBS concentrations were 0.35 mol/L and 7.0 mmol/L, respectively. With a further increase in the concentration of the monomer, the polymerization reaction proceeded too quickly, leading to greater difficulty in heat dissipation. This enhanced the chain transfer rate, ultimately resulting in a decrease in the polymer molecular weight. Based on the effects of the AM and AMBS concentrations on the *M_w_* values and the apparent viscosity of the synthesized copolymer, the optimum concentrations of AM and AMBS were 0.35 mol/L and 7.0 mmol/L, respectively.

It was obvious that the *M_w_* value of the synthesized copolymer decreased and the apparent viscosity of the solution increased with elevation of the MAPTAC concentration, which was distinct from the effects of the AM and AMBS concentrations (Figure 3c). This was because the neutralization between the cationic group in the MAPTAC molecule and the anionic group in the AMBS molecule in the weakly alkaline system reduced the copolymerization rate, which led to a decrease in the *M_w_* value of the copolymer. At low MAPTAC concentrations, the neutralization effect on the copolymerization rate was relatively weak, and such effect was strengthened by increasing the MAPTAC concentration. However, the apparent viscosity of the copolymer solution increased rapidly at first and then changed little with an increase in the MAPTAC concentration. This was attributed to the intra– and intermolecular association interactions of the synthesized copolymers, which altered the microstructure of the solution, regarding the viscosity, excess volume, and heat of mixing [18]. The increase in the MAPTAC concentration facilitated the enhancement of association interactions, thereby improving the apparent viscosity of the solution of the synthesized copolymer. Based on the effect of the MAPTAC concentration on the *M_w_* value and the apparent viscosity of P(AM/AMBS/MAPTAC), the optimal MAPTAC concentration was 1.0 mmol/L.

The effects of the initiator concentrations on the *M_w_* value and the apparent viscosity of P(AM/AMBS/MAPTAC) are shown in Figure 3d,e. The *M_w_* value and the apparent viscosity first increased, then decreased with an increase in the initiator concentration. When the AIBA concentration at the first stage was 0.03 wt%, the *M_w_* value and the apparent viscosity reached their maxima, at 24.52 mPa·s and 9.48 × 10^6^ g/mol, respectively. When the concentration of the redox initiation system at the second stage was 0.02 wt%, the *M_w_* value and the apparent viscosity were 24.92 mPa·s and 9.91 × 10^6^ g/mol, respectively. Based on the effect of the initiator concentration at the two stages on the *M_w_* value and the apparent viscosity of P(AM/AMBS/MAPTAC), the optimum concentrations of AIBA and the redox initiation system were 0.03 wt% and 0.02 wt%, respectively.

The *M_w_* value and the apparent viscosity of P(AM/AMBS/MAPTAC) first increased and then decreased as the polymerization temperature increased (Figure 3f). This is because the decomposition reaction of the initiator was inhibited at low temperatures, producing only a small amount of free radicals. As the polymerization temperature increased, the free radicals produced were sufficient to promote polymerization. When the polymerization temperature was 50 °C, the *M_w_* value and the apparent viscosity reached their maxima. As the polymerization temperature increased further, a large number of free radicals were produced within a short period, resulting in termination of chains and a reduction in molecular weight. The optimum polymerization temperature was 50 °C in this work.

### 3.2. Characterization of P(AM/AMBS/MAPTAC)

**Scheme 1:**
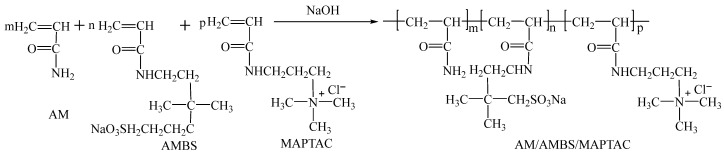
Synthesis route of zwitterionic polyacrylamide (P(AM/AMBS/MAPTAC)).

**Figure 4:**
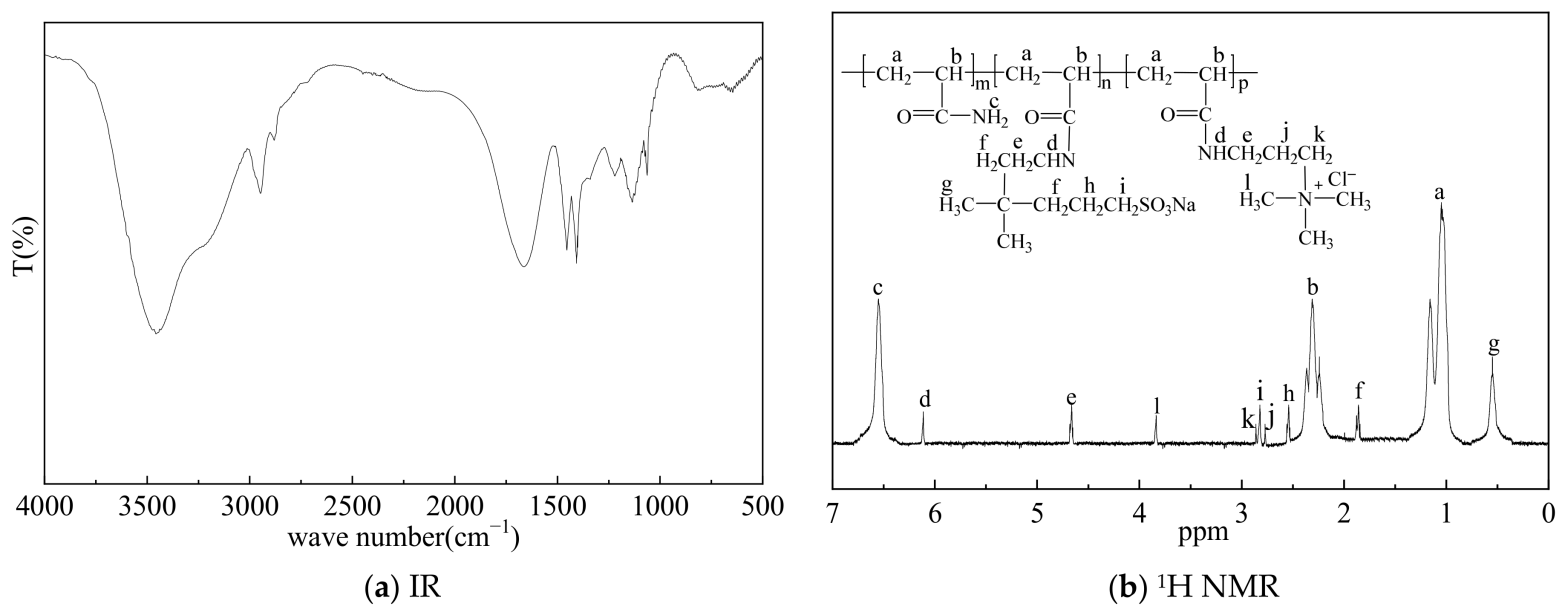
IR and ^1^H NMR spectra of P(AM/AMBS/MAPTAC).

P(AM/AMBS/MAPTAC) synthesized under the optimum polymerization conditions (Scheme 1) was characterized by FTIR and ^1^H NMR, and the results are shown in Figure 4.

The N–H stretching vibration peaks of the –CONH_2_ and –CONH– groups in the repeating units were found at about 3500 cm^−1^ in the IR spectrum (Figure 4a). The C=O stretching vibration peaks of the –CONH_2_ and –CONH– groups appeared at about 1640 cm^−1^. Meanwhile, the peaks of –NH– bending vibration were observed at about 1400 cm^−1^ and 1220 cm^−1^. The peaks at 1060 cm^−1^ and 650 cm^−1^ were attributed to S–O stretching vibration and C–S stretching vibration, respectively. Meanwhile, the peak of S=O asymmetric stretching vibration appeared at about 1130 cm^−1^. The peaks indicated that the monomers of AM, AMBS and MAPTAC were successfully copolymerized to obtain P(AM/AMBS/MAPTAC).

As shown in Figure 4b, the protons (a) of the –CH_2_– group in the polymeric chain appeared at 0.91–1.37 ppm, and the protons (b) of the –CH– group in the polymeric chain appeared at 2.13–2.59 ppm [19]. The peak at about 6.56 ppm was assigned to the protons (c) of the –CONH_2_ group in the AM unit. The protons (d) of the –CONH– group in the AMBS and MAPTAC units appeared at about 6.12–6.15 ppm. The peaks at 0.40–0.71 ppm were assigned to the protons (g) of the –CH_3_ group in the AMBS units. The protons (i) of the –CH– group combined with the –SO_3_^–^group in the unit AMBS appeared at about 2.83 ppm. The peaks at about 2.77 ppm and 2.87 ppm were assigned to the –CH_2_– protons of the –CONH–C–CH_2_–C–N– and –CONH–C–C–CH_2_–N– groups in the unit MAPTAC, respectively. The peak at about 4.65 ppm was assigned to the protons of the –CH_2_– group combined with the –CONH– group in the AMBS and MAPTAC units. The peak at about 3.84 ppm was assigned to the protons of the –CH_3_ group in the MAPTAC units. Moreover, the integral area ratio of all the protons was consistent with the chemical structure, and it agreed well with the IR data, confirming the successful synthesis of P(AM/AMBS/MAPTAC).

### 3.3. Rheological Properties of P(AM/AMBS/MAPTAC) Solution

**Figure 5:**
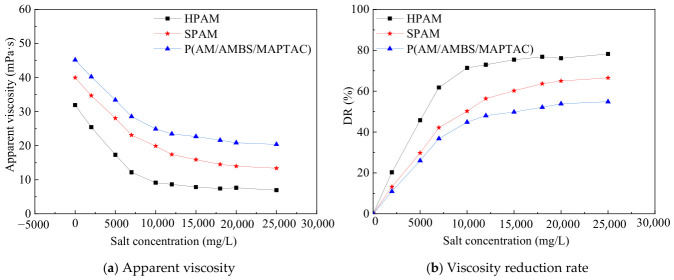
The effect of salinity on the viscosity stability of the polymer solutions.

The salt tolerance of polymer solutions (2000 mg/L) was studied at 60 °C and a shear rate of 150 s^−1^ when the mass ratio of NaCl, CaCl_2_ and MgCl_2_ was 60:4:3 (Figure 5); the results showed that the apparent viscosity of the three polymer solutions decreased with increasing salinity. This was because the complexation between the cations (Na^+^, Ca^2+^ and Mg^2+^) and the polymer led to charge shielding of the copolymer, which led to the copolymer backbone shrinking in the presence of salt [20]. In polymer solutions, intrachain complexation occurs at low salinity, intrachain and interchain complexation take place at medium salinity, and interchain complexation occurs at high salinity [21], which reduces the apparent viscosity of the copolymer solution. The apparent viscosity of the copolymers with sulfonic acid groups (P(AM/AMBS/MAPTAC) and SPAM) was higher than that of HPAM with carboxylic acid groups, and the apparent viscosity of the P(AM/AMBS/MAPTAC) solution with zwitterionic groups was higher than that of SPAM (Figure 5a). Moreover, the P(AM/AMBS/MAPTAC) solution had good viscosity stability in the high–salinity system compared with HPAM and SPAM (Figure 5b). When the salinity was 10,000 mg/L and the shear rate was 150 s^−1^, the apparent viscosity for the P(AM/AMBS/MAPTAC) solution was 24.92 mPa·s and the viscosity reduction rate was 44.86% at 60 °C for 30 d. Nevertheless, under the same test conditions and mass concentration of the polymer, the apparent viscosity and viscosity reduction rate of the HPAM solution were 9.12 mPa·s and 71.43%, respectively. This was because complexation between the carboxylic acid groups and metallic ions reduced the solubility of HPAM, while the sulfonic acid groups had excellent salt tolerance. For P(AM/AMBS/MAPTAC) containing both sulfonic acid groups and quaternary amine groups, intermolecular hydrophobic associations occurred and inhibited intrachain and interchain complexation, which improved the salt tolerance of the polymer [10].

**Figure 6:**
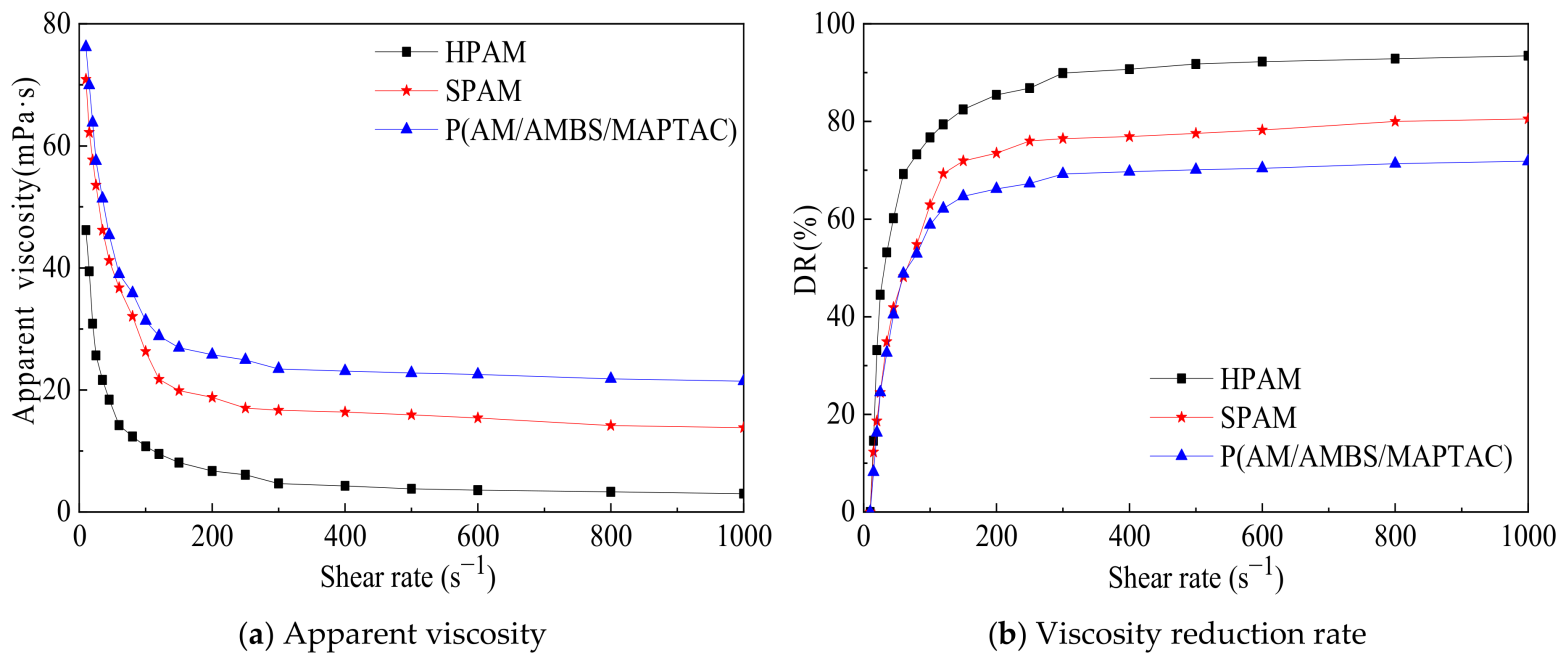
The effect of shear rate on the viscosity stability of the polymer solutions.

The shear resistance of the polymer solutions (2000 mg/L) was studied at 60 °C when the salinity was 10,000 mg/L (Figure 6), and the results showed that the apparent viscosity of all three polymer solutions decreased with increasing shear rate. This phenomenon is because the polymer chains form physical crosslinks through mutual entanglement or van der Waals interactions, and the crosslinks undergo continuous disintegration and reconstruction under molecular thermal motion [22]. However, it is worth noting that the apparent viscosity of the three polymer solutions followed the trend P(AM/AMBS/MAPTAC) > SPAM > HPAM at the same shear rate (Figure 6a). The P(AM/AMBS/MAPTAC) solution exhibited higher apparent viscosity and better viscosity stability at a high shear rate compared with HPAM and SPAM (Figure 6). When the salinity was 10,000 mg/L and the shear rate was 300 s^−1^, the apparent viscosity of the P(AM/AMBS/MAPTAC) solution was 23.45 mPa·s and the viscosity reduction rate was 69.23% at 60 °C for 30 d. Nevertheless, under the same test conditions and polymer mass concentration, the apparent viscosity and the viscosity reduction rate of the HPAM solution were 4.66 mPa·s and 89.91%, respectively. This probably indicates that the zwitterionic group of P(AM/AMBS/MAPTAC) improved the shear resistance and apparent viscosity of the polymer solution because of the electrostatic interactions between the zwitterionic segments. The synergistic effects of zwitterionic electrostatic interactions and hydrophobic associations in P(AM/AMBS/MAPTAC) help maintain a higher level of entanglement, resulting in a slower decrease in apparent viscosity compared to the other two polymers. This excellent shear resistance is crucial for polymer flooding applications: the polymer solution is subjected to high shear forces during injection into reservoirs, and this property ensures that the solution retains sufficient viscosity to effectively displace oil.

**Figure 7:**
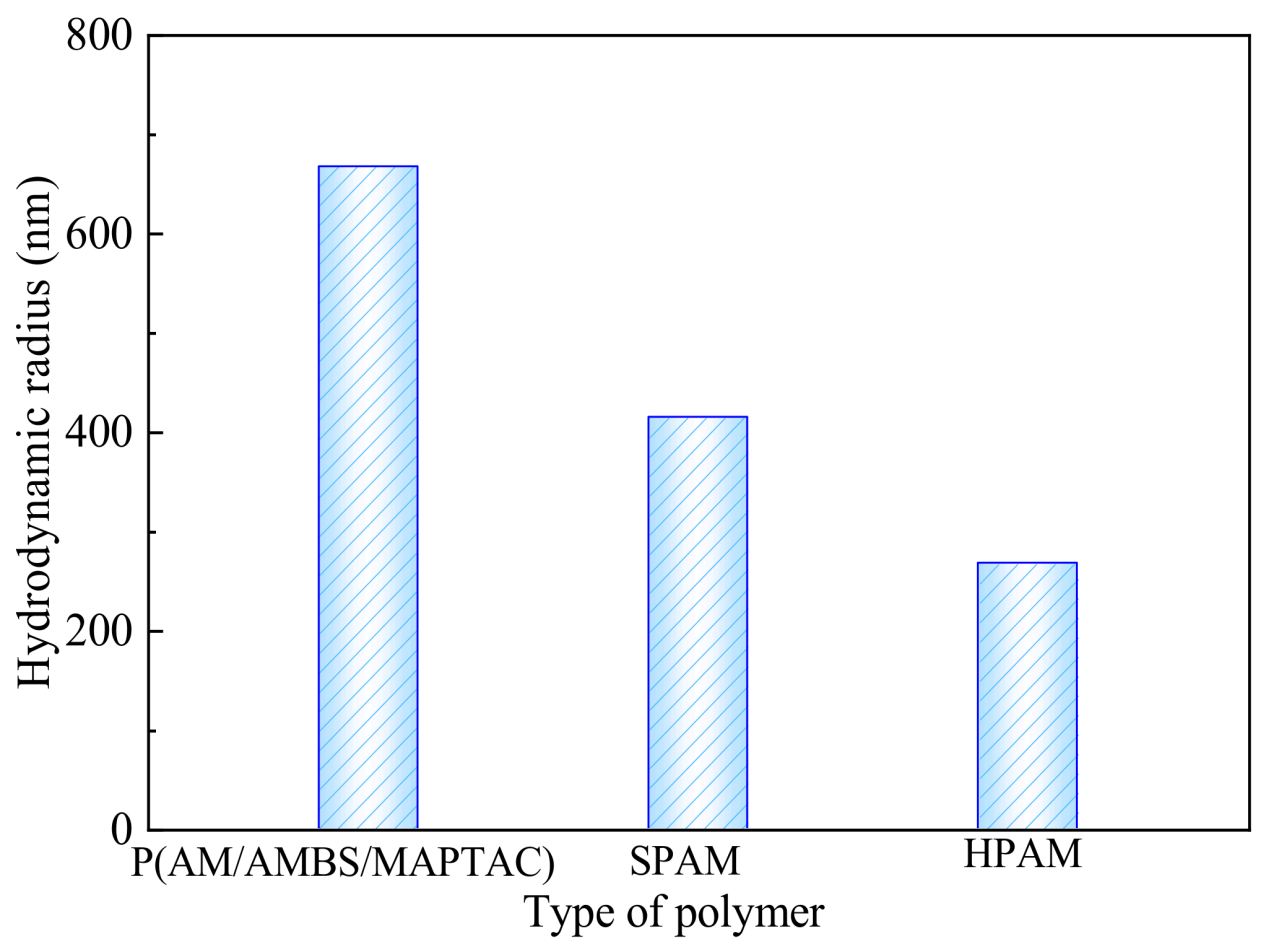
Hydrodynamic radii of different polymer solutions at a polymer concentration of 2000 mg/L.

To further represent the aggregate sizes of the polymer molecules in solution, the hydrodynamic radii of different polymer solutions at a polymer concentration of 2000 mg/L were measured [9], and the results are shown in Figure 7. The hydrodynamic radius of P(AM/AMBS/MAPTAC) was greater than those of SPAM and HPAM, which further confirmed that the zwitterionic electrostatic interactions and hydrophobic associations promoted molecular entanglement because of the zwitterionic segments of the P(AM/AMBS/MAPTAC) molecules. The findings further validated the enhanced apparent viscosity (Figure 5a and Figure 6a).

### 3.4. Emulsification Performance of P(AM/AMBS/MAPTAC)

**Figure 8:**
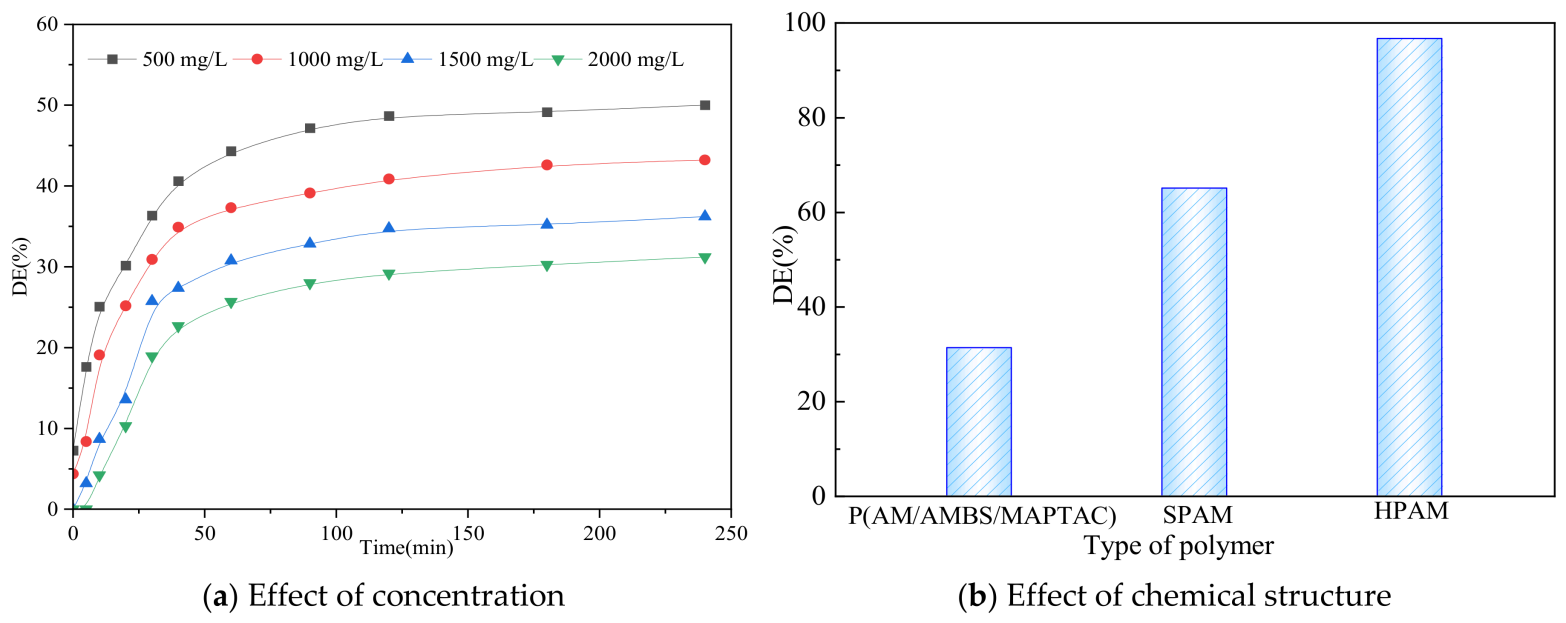
Emulsification properties of the polymers.

The emulsification performance of P(AM/AMBS/MAPTAC) at various concentrations was assessed at a salinity of 5000 mg/L and 60 °C (Figure 8a). As clearly shown in Figure 8a, the emulsification performance of P(AM/AMBS/MAPTAC) was positively correlated with the polymer concentration and negatively correlated with time. Increasing the polymer concentration was favorable for emulsifying oil in the polymer solution, which was because the polymer molecules had two roles in the simulated emulsion. On the one hand, the polymer could adsorb at the oil–water interface to reduce the interfacial tension, thereby promoting the formation and stabilization of emulsion droplets. On the other hand, the polymer chains could form a three-dimensional network structure in the solution, preventing the aggregation and coalescence of emulsion droplets by increasing the viscosity of the continuous phase. When the polymer concentration was low, an insufficient number of polymer molecules accumulated at the interface, and the network structure formed in the solution was not dense enough, resulting in a relatively poor emulsification effect. As the concentration increased, more polymer molecules participated in interfacial adsorption and network formation, leading to a significant improvement in emulsification performance. However, when the concentration exceeded a certain threshold, the improvement in emulsification performance tended to level off, which may be attributed to saturated interfacial adsorption and the limited increase in solution viscosity caused by excessive polymer entanglement.

In order to further understand the emulsification behavior of P(AM/AMBS/MAPTAC) with sulfonic acid and quaternary amine groups, the emulsification performance of HPAM and SPAM at a salinity of 5000 mg/L and a polymer concentration of 2000 mg/L was investigated at 60 °C for 4 h (Figure 8b). The simulated emulsion formed from P(AM/AMBS/MAPTAC) exhibited a lower DE value than the simulated emulsions formed from HPAM and SPAM, indicating that the former had better emulsification stability. This phenomenon was attributed to the synergistic effect of the sulfonic acid and quaternary amine groups in P(AM/AMBS/MAPTAC). The sulfonic acid groups enhanced the hydrophilicity of the polymer, while the quaternary amine groups improved the adsorption capacity of the polymer molecules at the oil–water interface, enabling more effective reduction of the interfacial tension. Additionally, the molecular weight and the charge density of the polymer also played crucial roles. Higher molecular weight may lead to stronger steric hindrance, preventing the coalescence of emulsion droplets. An appropriate charge density balances the electrostatic repulsion between droplets, further maintaining the stability of the emulsion system.

**Figure 9:**
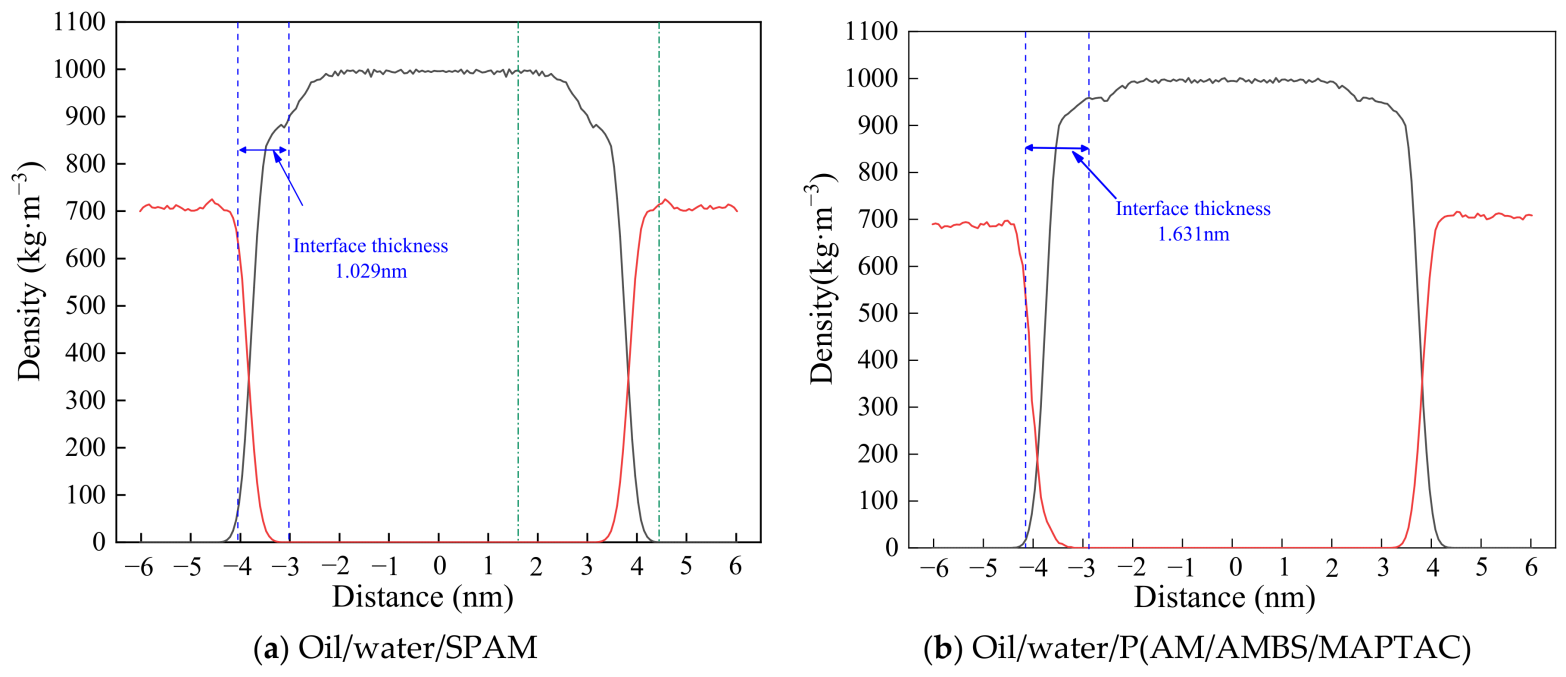
Z-direction density distribution of oil/water/copolymer systems.

To investigate the effects of SPAM and P(AM/AMBS/MAPTAC) at the oil–water interface, molecular dynamics simulations were performed, and the results are shown in Figure 9.

The oil–water interface thicknesses of the SPAM system and the P(AM/AMBS/MAPTAC) system (Figure 9) were 1.029 nm and 1.631 nm, respectively. An increase in interface thickness indicates an improvement in the adsorption properties at the oil–water interface. Compared with SPAM, P(AM/AMBS/MAPTAC) has stronger lipophilicity and surface distribution at the oil–water interface, which increases the interface thickness to enhance the emulsification performance. P(AM/AMBS/MAPTAC), with its amphiphilic structure, is conducive to better emulsification properties.

### 3.5. Oil Displacement Performance of P(AM/AMBS/MAPTAC)

**Figure 10:**
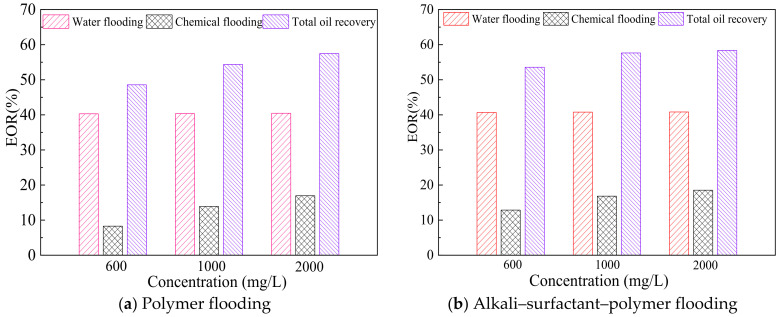
The effect of the P(AM/AMBS/MAPTAC) concentration on the different flooding systems.

For a salinity of 5000 mg/L and a temperature of 60 °C, the effects of the concentration of P(AM/AMBS/MAPTAC) on the oil displacement efficiency for the polymer flooding system and the alkali–surfactant–polymer flooding system are shown in Figure 10. The results of the oil displacement experiment indicated that P(AM/AMBS/MAPTAC) was favorable for enhancing oil recovery at 60 °C (Figure 10). For the polymer flooding, the chemical oil recovery increased from 8.26% to 16.95% and the total oil recovery increased from 48.56% to 57.48% with an increase in the polymer concentration from 600 mg/L to 2000 mg/L (Figure 10a). For the alkali–surfactant–polymer flooding, the chemical oil recovery increased from 12.87% to 18.54% and the total oil recovery increased from 53.57% to 58.36%. This phenomenon can be attributed to the fact that a high concentration of polymer increases the viscosity of the flooding system, which effectively improves the sweep efficiency by reducing the mobility ratio between the displacing fluid and the crude oil [5]. Additionally, the enhanced viscoelasticity of the polymer solution at higher concentrations effectively plugs the high–permeability zones, forcing the displacing fluid to flow into the low-permeability areas that were previously unswept, thereby increasing the overall oil recovery.

Compared with that for polymer flooding (Figure 10a), the increase in the chemical oil recovery for alkali–surfactant–polymer flooding was relatively smaller (Figure 10b). This was because the alkali–surfactant–polymer system combined the macroscopic volumetric sweep efficiency improvement from the polymer reducing the water–oil mobility ratio with the ability of the surfactant to enhance the microscopic sweep efficiency [23]. This enhancement resulted from the dramatic reduction in the oil–water interfacial tension increasing the capillary number by orders of magnitude, leading to the required range for efficient oil recovery. Alkali formed the surface–active substance by reacting with organic acid in the crude oil, and this substance interacted synergistically with the added surfactants to produce ultra–low interfacial tension [24]. The addition of alkali to the surfactant system changed the adsorption behavior of the surfactant molecules at the oil–water interface, enhancing the stability of the interface film. Adding the polymer increased the viscosity of the aqueous phase, decreasing the aggregation rate of the emulsion droplets. Owing to the synergy of these three components, alkali–surfactant–polymer flooding is widely applied in both pilot and field operations with the objective of achieving optimal chemical performance for large injection volumes at minimum cost.

**Figure 11:**
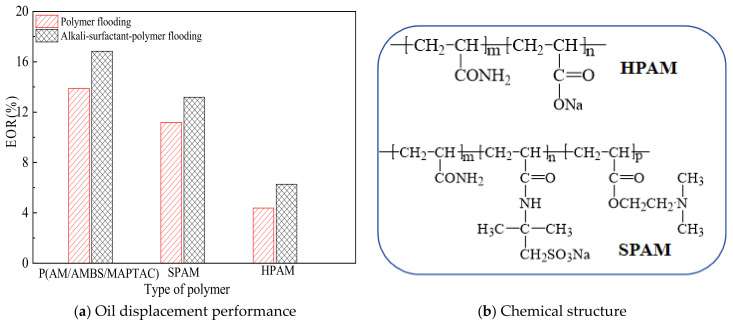
The effect of the chemical structures of polymers on the oil displacement efficiency.

The effects of chemical structure on the oil displacement efficiency of the polymer flooding system and the alkali–surfactant–polymer flooding system are shown in Figure 11a. Compared with those with HPAM and SPAM (Figure 11b), both the polymer flooding and the alkali–surfactant–polymer flooding formed with P(AM/AMBS/MAPTAC) exhibited higher oil displacement efficiency. Compared with those for HPAM, the enhanced oil recovery (EOR) values of the polymer flooding system and the alkali–surfactant–polymer flooding system containing P(AM/AMBS/MAPTAC) were increased by 9.51% and 10.57%, respectively (Figure 11a). The excellent chemical oil recovery performance of P(AM/AMBS/MAPTAC) is probably attributed to two roles. On the one hand, the P(AM/AMBS/MAPTAC) solution has higher viscosity and better viscosity stability under high–temperature and high–salinity conditions compared with HPAM and SPAM (Figure 5 and Figure 6). This is because the intermolecular hydrophobic associations of P(AM/AMBS/MAPTAC) with sulfonic acid groups and quaternary amine groups inhibit the curling and entanglement of the polymer molecular chains. On the other hand, the extended–coil structure of P(AM/AMBS/MAPTAC) and the space steric hindrance from hydration effectively improve the adsorption capacity of the polymer molecules at the oil–water interface and the viscoelasticity of the oil–water interface film (Figure 9), thereby improving the chemical oil recovery (Figure 11a). Moreover, the emulsion formed of P(AM/AMBS/MAPTAC) with zwitterionic groups has higher stability than other emulsions (Figure 8b), which is favorable for achieving higher ultimate oil recovery.

## 4. Conclusions

The zwitterionic copolymer P(AM/AMBS/MAPTAC) was synthesized via segmentation initiation using AM, AMBS and MAPTAC as monomers. P(AM/AMBS/MAPTAC) with sulfonic acid groups and quaternary amine groups exhibited outstanding salt tolerance and shear resistance. Compared with HPAM and SPAM with higher *M_w_*, the P(AM/AMBS/MAPTAC) solution had higher apparent viscosity even with a lower *M_w_* value and exhibited better viscosity stability. Moreover, P(AM/AMBS/MAPTAC) exhibited higher emulsion stability and a thicker oil–water interface than HPAM and SPAM, owing to the synergistic effect of the sulfonic acid groups and quaternary amine groups in P(AM/AMBS/MAPTAC). Both the polymer flooding system and the alkali–surfactant–polymer flooding system containing P(AM/AMBS/MAPTAC) had high chemical oil recovery, and the oil displacement efficiency was higher than that of HPAM and SPAM in their corresponding polymer flooding and alkali–surfactant–polymer flooding systems. As a result, this study offers significant guiding value for the development of techniques focused on enhancing oil recovery.

## Figures and Tables

**Table 1:** The effect of the anionic monomer type on the properties of the copolymer.

Anionic Monomer	M_w_ × 10^6^ (g/mol)	Apparent Viscosity (mPa∙s)
AA	3.16	8.32
AMPS	9.99	19.10
AMBS	9.73	21.14

**Table 2:** The effect of the cationic monomer type on the properties of the copolymer.

Cationic Monomer	M_w_ × 10^6^ (g/mol)	Apparent Viscosity (mPa∙s)
DMAEMA	9.73	21.14
DMAPMA	9.25	22.03
DAC	8.69	23.05
MAPTAC	9.15	23.96

## Data Availability

The original contributions presented in this study are included in the manuscript. Further inquiries can be directed to the corresponding authors.
